# Risk factors for stress in professionals at the North emergency care unit in Palmas, Tocantins, Brazil

**DOI:** 10.47626/1679-4435-2022-782

**Published:** 2023-02-13

**Authors:** Tiago Veloso Neves, Neumar Inácio Martins Campos Junior, João Albino Neto

**Affiliations:** 1 Instituto Tocantinense Presidente Antônio Carlos, Curso de Medicina, Palmas, TO, Brazil; 2 Centro de Referência em Saúde do Trabalhador, Secretaria Municipal de Saúde de Palmas, Palmas, TO, Brazil

**Keywords:** occupational stress, occupational health, emergency medical services, estresse ocupacional, saúde do trabalhador, serviços médicos de emergência

## Abstract

**Introduction:**

Emergency care units provide intermediate complexity care services, are open 24 hours a day, and are frequently required to attend to high levels of demand, especially so during the Covid-19 pandemic. On-duty shift work at emergency care units is highly conducive to causing excessive stress.

**Objectives:**

To identify the risk factors for excessive stress among workers at the North emergency care unit, in Palmas, Tocantins, Brazil.

**Methods:**

A questionnaire was administered covering basic information and data on lifestyle to the workers at the unit, in addition to the Epworth Sleepiness Scale and a single-item instrument for diagnosis of stress.

**Results:**

It was possible to recruit 44 participants. It was observed that 57% of the participants exhibited stress and 31.82% had excessive sleepiness. Having more than one job, drinking alcohol, having attended higher education, and having excessive sleepiness increased the likelihood of exhibiting stress. There was a statistical significant association of very large magnitude between performing housework and exhibiting symptoms of stress (p = 0.028; r_ɸ_ = 0.36).

**Conclusions:**

The high percentage of workers with stress found among the study participants reveals a need for measures to review their working processes, such as creating space for dialogue between workers and management or implementing shared management, with the objective of minimizing development of work-related disorders, with benefits both for the workers and the Unit.

## Abstract (pt)

**Introdução:**

As unidades de pronto atendimento prestam serviços de complexidade intermediária, operando em regime de plantão (24 horas) e atendendo frequentemente a altas demandas, especialmente no período da pandemia por covid-19. Serviços de pronto atendimento em regime de plantão são muito propícios a produzir estresse excessivo em seus trabalhadores.

**Objetivos:**

Verificar quais são os fatores de risco para estresse excessivo entre trabalhadores da unidade de pronto atendimento Norte, em Palmas, Tocantins.

**Métodos:**

Foi aplicado um questionário acerca de informações básicas e de estilo de vida dos trabalhadores dessa unidade, além da Escala de Sonolência de Epworth e um instrumento de um único item para diagnóstico do estresse.

**Resultados:**

Foi possível obter a adesão de 44 participantes. Constatou-se que 57% dos participantes apresentavam estresse, e 31,82% apresentavam sonolência diurna excessiva. Ter duplo vínculo, ser etilista, ser profissional de nível superior e ter sonolência diurna excessiva aumentaram as chances de apresentar estresse. Houve associação estatística significativa e de muito alta magnitude entre realizar tarefas domésticas e apresentar sintomas de estresse (p = 0,028; r_ɸ_ = 0,36).

**Conclusões:**

O alto percentual de servidores com estresse encontrado entre os participantes suscita medidas de revisão do processo de trabalho, tais como promover espaços de diálogo entre servidores e gestores ou promover gestão compartilhada, no intuito de minimizar o desenvolvimento de transtornos relacionados ao trabalho e beneficiar tanto os servidores quanto o próprio Serviço.

## INTRODUCTION

An emergency care unit (UPA, from “*unidade de pronto atendimento*”, in Portuguese) is part of the Brazilian healthcare strategy, offering intermediate complexity services, with facilities for X-rays, electrocardiography, pediatrics, laboratory tests, and observation beds. Emergency care units are open 24 hours per day, worked in three shifts: morning, afternoon/evening, and night. The size of the team varies according to the area covered.^1^ Emergency care units provide a very important service to the community. The professional team at a UPA is made up of physicians, dentists, nurses, and nursing technicians, who perform a variety of tasks, with differing workloads. Over time, these workloads can cause non-physiological stress.

Stress is an adaptive physiological response to adverse situations, triggered naturally by the body to enable appropriate responses to a threat or difficulty. However, a prolonged state of stress becomes pathogenic.^2,3^

An earlier study investigated stress among the professionals working at the UPAs in Palmas, Tocantins (TO), Brazil, using the Perceived Stress Scale (PSS), finding that there were workers with elevated stress levels among the professionals with higher, technical, and secondary-level education, although the highest mean level was observed among the professionals with higher education (17.8 ± 6.7).^4^ However, that study did not test whether there were statistical associations between the variables described.

Since the “Factors associated with lumbar pain in health professionals at the North Urgent Care Unit in Palmas - Tocantins” study also found that stress was prevalent among the workers at the North UPA and it became clear that there was a need to study the relationships between stress and the other study variables in a separate analysis, there is an interest in discussing stress among the professionals working at the North UPA and the factors associated with it.

## METHODS

This was a cross-sectional observational study derived from the “Factors associated with lumbar pain in health professionals at the North Urgent Care Unit in Palmas - Tocantins” project that was carried out in the same municipal district and approved by the Research Ethics Committee at the Fundação Escola de Saúde Pública (FESP), decision number 3.990.631. In view of the large volume of data collected, it was considered better to conduct a separate analysis focused on stress among these professionals and the risk factors for it.

The study population comprised health professionals assigned to the North UPA (NUPA) in Palmas, TO, Brazil, at Quadra 203 Norte, Avenida LO - 06, APM 02, and the data were collected from April to June of 2020. This unit is the only UPA responsible for the North region of the municipal district of Palmas. The inclusion criteria for the study were being a healthcare worker assigned to the NUPA and agreeing to take part, signing a free and informed consent form, in accordance with National Health Council (Conselho Nacional de Saúde- CNS) Resolution 466/2012. Workers were only excluded from the study if they were on holiday or on leave during the data collection period, had suffered a spinal trauma unrelated to their jobs during the 3 months preceding the study, or had undergone a surgical procedure on the spinal column during the 3 months preceding the study.

According to information provided by Human Resources, there were 157 workers assigned to the North UPA at the time of the study. The sample size calculation estimated that 112 participants would be needed for a 95% confidence interval (95%CI) and 5% margin of error. Data were collected using questionnaires delivered to workers while they were on duty. These instruments covered personal, socioeconomic, and cultural data and also contained questions related to their medical histories. Despite these precautions, it was difficult to enroll the workers.

The participants were assessed for presence of stress using the following question: “Stress is our bodies’ physical response to a stimulus. When stressed, a person becomes tense, restless, nervous, and anxious and has insomnia, because the mind is constantly unbalanced. Have you experienced this type of stress continuously for 7 successive days or more during the last 12 months?” This question has been validated by Elo et al.^5^ as an instrument for assessment of symptoms of stress and offers the response options “yes” or “no”.

The Epworth Sleepiness Scale (ESS) was also used to assess participants’ sleep quality. This questionnaire contains six questions that assess the possibility of a person dozing during daily activities such as watching television, sitting quietly, waiting in traffic, and others. The score varies from 0 to 24, and scores over 10 indicate a diagnosis of excessive daytime sleepiness.^6,7^

Data were input to Microsoft Excel spreadsheets. Next, measures of central tendency were extracted, expressing the results as absolute and relative frequencies, in graphs and tables. Epi Info version 7.2.4.0 (Centers for Disease Control, Atlanta, United States) and BioEstat version 5.3 were used to tabulate data and conduct statistical analyses.^8^ Odds ratios (OR) and Fisher’s exact test were used to analyze relationships between the variables. The phi (ɸ) coefficient was used to determine the magnitude of associations between variables. The coefficient was interpreted using the Akoglu criteria:^9^ > 0.25: very strong; > 0.15: strong; > 0.10: moderate; > 0.05: weak; > 0: absent or very weak.

## RESULTS

At the end of data collection, it had proven possible to recruit 44 participants, which constitutes a sample 60.71% smaller than the planned sample size. However, judging by the effect size estimated by the phi coefficient, this loss does not appear to have affected the associations between variables. With regard to the sociodemographic data, 79.55% of the participants were female, and 56.82% had brown skin color. In relation to the age group of interviewees, 45.45% were aged from 26 to 35 years. Additionally, 97.73% were non-smokers and 62.50% used a car as means of transport (Table 1).

**Table 1:** Sociodemographic characteristics of professionals at the North emergency care unit (UPA), Palmas (TO), Brazil

Variable	Absolute frequency (n)	Relative frequency (%)
Sex		
Male	9	20.45
Female	35	79.55
Skin color		
White	12	27.27
Black	6	13.64
Yellow	1	2.27
Brown	25	56.82
Age group (years)		
18 to 25	3	6.82
26 to 35	20	45.45
36 to 45	13	29.55
46 to 55	7	15.91
56 to 60	1	2.27
Marital status		
Single	16	36.36
Married	23	52.27
Stable relationship	1	2.27
Divorced	3	6.82
Widowed	1	2.27
Children		
None	14	31.82
1 or 2	20	45.45
3 or 4	10	22.73
5 or 6	0	0.00
More than 6	0	0.00
Means of transport ^*^		
Car	25	62.50
Motorbike	10	25.00
Bus	5	12.50
Smoker		
Yes	1	2.27
No	43	97.73
Drinks alcohol		
Yes	19	43.18
No	25	56.82
Physical exercise (times per week)		
0	16	36.36
1 to 2	9	20.45
3 to 5	17	38.64
6 to 7	2	4.55

* Some participants did not answer this question.

In turn, the occupational data shown in Table 2 reveal that the majority of the participants were nursing professionals; 50% of the sample were nursing technicians and 22.73% were nurses, while 56.82% had been working in the profession for more than 10 years. Overall, 72.73% of the sample were permanent staff, while 90.91% also performed housework.

**Table 2:** Occupational data of professionals at the North emergency care unit (UPA), Palmas (TO), Brazil

Variable	Absolute frequency (n)	Relative frequency (%)
Occupation		
Health services assistant	1	2.27
Social worker	3	6.82
Biomedical specialist	1	2.27
Dentist	1	2.27
Nurse	10	22.73
Physician (female)	3	6.82
Physician (male)	1	2.27
Nursing technician	22	50.00
Radiology technician	2	4.55
Time in the profession (years)		
< 1	3	6.82
1 to 3	3	6.82
4 to 6	11	25.00
7 to 10	2	4.55
More than 10	25	56.82
Time working at the emergency care unit		
0 to 12 months	13	29.54
1 to 5 years	16	36.36
6 to 10 years	10	22.73
More than 10 years	5	11.36
Working hours per week		
20	7	15.91
30	32	72.72
40	4	9.09
60	1	2.27
Employment status		
Contract worker	10	22.73
Permanent worker	32	72.73
On secondment	2	4.55
Other job/attachment		
Yes	21	47.73
No	23	52.27
Performs housework?		
Yes	40	90.91
No	4	9.09

Analyzing the results of the validated instruments that had been administered, it was found that 25 of the 44 participants (57%) exhibited symptoms of stress at the time they answered the questionnaires, while 14 (31.82%) participants scored more than 10 points on the ESS, characterizing excessive sleepiness. Overall, the participants had a mean ESS score of 8.6 points, with a median of 9 points and a standard deviation (SD) of ± 3.79.

Table 3 shows the results of the analysis of associations between the study variables and presence of stress symptoms. Despite the researchers’ initial hypotheses, having children, working more than 30 hours a week, or having worked in the NUPA for more than 5 years did not significantly increase the likelihood of participants having stress symptoms at the time of data collection. There were also no differences between men and women in the likelihood of stress, although the OR for men was slightly lower than the OR for women. However, being single and being physically active appear to have reduced the likelihood of stress, as did being a contract worker, which the researchers found particularly surprising since career stability is normally considered a factor that reduces workers’ vulnerability to work-related mental disorders. Although none of these three variables attained statistical significance, they all exhibited a strong magnitude of association with the outcome stress (r_ɸ_ > 0.15).

**Table 3:** Associations between study variables and stress in professionals at the North emergency care unit (UPA), Palmas (TO), Brazil

Variable	Odds ratios	95%CI	p	r_ɸ_
Sex				
Male	0.94	0.21-4.1	1.00	0.01
Female				
Marital status				
Single	0.5	0.13-1.82	0.34	0.16
Married				
Children				
Yes	1.5	0.41-5.38	0.74	0.09
No				
Physical activity				
Yes	0.45	0.12-1.65	0.34	0.18
No				
Housework				
Yes	-	-	0.03^*^	0.36
No				
Smoking				
Yes	-	-	1.00	0.13
No				
Drinks alcohol				
Yes	3.56	0.98-12.96	0.07	0.29
No				
Abnormal sleepiness				
Yes	4.19	0.97-18.12	0.06	0.3
No				
Educational level				
Higher education	3.56	0.98-12.96	0.07	0.29
Secondary education				
Employment status				
Contract worker	0.31	0.06-1.47	0.25	0.24
Permanent employee				
More than one job				
Yes	2.18	0.64-7.40	0.21	0.19
No				
Hours worked per week				
More than 30	1.16	0.17-7.73	1.00	0.02
Up to 30				
Time in the profession (years)				
More than 10	1.98	0.58-6.66	0.36	0.16
Less than 10				
Time working at the UPA (years)				
More than 5	0.81	0.23-2.83	0.76	0.05
Less than 5				

* p < 0.05, statistically significant.

95%CI = 95% confidence interval; r_ɸ_ = phi coefficient.

In contrast, having more than one job, drinking alcohol, and having a higher education qualification all considerably increased the likelihood of having symptoms of stress for 7 successive days or more. Scoring over the cutoff on the ESS (> 10) increased the likelihood of stress by more than four times. However, none of the variables above had a statistically significant association with stress, which is possibly related to the small size of the sample that it was possible to recruit, since sample size can affect p value. However, observing the magnitudes of the associations between these variables and stress, it can be seen that all of them exhibited strong (r_ɸ_ > 0.15) or very strong associations (r_ɸ_ > 0.25). Nonetheless, the obligation to perform housework was significantly associated with symptoms of stress and had the largest magnitude of association (r_ɸ_ = 0.36). The magnitudes of the other variables’ associations with stress were classified as moderate, weak, or very weak.

It was not possible to calculate the OR for stress associated with smoking or with performing housework because there were zeros in the contingency tables for these variables.

## DISCUSSION

In common with the study by Mota et al.,^4^ we were able to confirm that a high number of health professionals had symptoms of stress. In our study, the group with stress accounted for more than half of the healthcare workers. This is compatible with the scientific evidence, since several different studies have shown that on-duty shift work affects homeostasis and is associated with development of a series of pathological conditions, including excessive stress.^3,10-15^ This is understandable, taking into account the inherent features of the routine of a UPA: the sometimes excessive workloads, the high emotional and physical demands, the biological risk, the prejudice to sleep quality caused by shift working and, specifically during 2020 when data were collected, the need to be on the front line of caring for suspected and confirmed cases of Covid-19, intensifying the pressure and workload.

The excessive sleepiness affecting 31.82% of the sample corroborates published data that list this as one of the more common conditions related to on-duty shift work (night shifts, especially) and this is a factor that is frequently associated with stress or identified as a risk factor.^12,14,16,17^ One study found that people who exhibited moderate to severe stress levels had a three times greater likelihood of also having excessive sleepiness, showing that there is a link between stress and poor sleep quality.^18^

The fact that the contract workers had a lower likelihood of having stress symptoms than the permanent workers may have been influenced by the size of the sample, which contained a majority of permanent staff, since it is normally considered that contract employees are more susceptible to stress because they do not have stable employment status. However, the magnitude of this correlation, expressed by r_ɸ_ = 0.24, suggests that there is a strong correlation in this association, and since r_ɸ_, is an estimate of effect size, it is subject to little influence from sample size, in contrast with the p value.^9^

The “marital status” variable showed that single workers are at least 50% less susceptible to symptoms of stress than married workers, without a statistically significant association, but with a strong magnitude. However, the association that was found between performing housework and symptoms of stress had an even larger magnitude of association. This leads us to consider that it may not be the commitments of matrimony itself that make participants susceptible to stress, but the fact that they do shift work at a 24-hour unit, with all the challenges of a UPA, during the pandemic, and are still responsible for domestic chores when they get home. Sometimes, marriage itself may act as a protective factor against stress.^17^

It was to be expected that the likelihood of stress would increase significantly the longer the time in the profession, the longer time working at the UPA, and the longer the working hours, but this was not objectively confirmed among our participants. It is possible that the more experienced workers in this group had developed strategies for coping with the routine and demands of the UPA, becoming more resilient.

Although it used different instruments, this study also confirmed the finding reported by Mota et al.^4^ that professionals with higher education are more susceptible to perception of symptoms of stress. This may be because of the fact that these professionals are responsible for more complex decisions and processes within clinical management, demanding greater cognitive effort.

## CONCLUSIONS

This study confirmed the findings in the literature on the presence of stress among shift workers and also the relationship between excessive sleepiness and stress, through which the former increases the likelihood of developing the latter by more than four times. Having more than one job and drinking alcohol more than doubled the likelihood of symptoms of stress. It was also found that performing housework is significantly associated with this condition. Although the sample size was insufficient for generalization of the results, it is possible to confirm that the aforementioned variables are factors associated with development of stress among workers at the NUPA.

The workplace can be a source of both pleasure and suffering and sickness. To make the workplace less pathogenic, it is necessary to open spaces for discussion to reveal workers’ anxieties, promote shared management, and operate the work logistics in such a way as to avoid overloading workers, among other measures. This study should enable management to identify points to be addressed for the good of the workers’ health and for the good of the Unit itself.
